# Supplementary material for: H2-M and H2-O as Targeting Vehicles for the MHC Class II Processing Compartment Promote Antigen-Specific CD4+ T Cell Activation
Source: Vaccines (Basel). 2021 Sep 22;9(10):1053. doi: 10.3390/vaccines9101053 (PMC8540253; doi:10.3390/vaccines9101053)
Supplement: Supplementary file 1 [file vaccines-09-01053-s001.zip › vaccines-1328914-supplementary.pdf]

S1. Supplementary material Lapazio et al

Sequences of the H2-M and H2-O OVA fusion constructs

>H2-Ma-OVA short

meheqksgavllrllrllwllphswavlGGGGSADQARELINSWVESQTNGIIRNVLQPSSVDSQTAMVLVNAIVFKGLWEKT
FKDEDTQAMPFRVTEQESKPVQMMYQIGLFRVASMASEKMKILELPFASGTMSMLVLLPDEVSGLEQLESIINFEKLTEWTSS
NVMEERKIKVYLPRMKMEEKYNLTSVLMAMGITDVFSSSANLSGISSAESLKISQAVHAAHAEINEAGREVVGSAEAGVDAAS
VSEEFRADHPFLFCIKHIATNAVLFFGRCVSPGGGGSEQKLISEEDLGGGGSnalcgvafglgvlgtimgivfflcsqrpcsg
d

>H2-Ma-OVA long

meheqksgavllrllrllwllphswavleastpvfwddpqnhtfrhtlfcqdgipniglsetydedelfsfdfsqntrvprlp
dfaewaqgqgdasaiafdksfcemlmrevspklegqipvsrglpvaevftlkplefgkpntlvcfisnlfpptltvnwqlhsa
pvegasptsisavdgltfqafsylnftpepfdlysctvtheidrytaiaywvpqnalpsdllenaGGGGSADQARELINSWVE
SQTNGIIRNVLQPSSVDSQTAMVLVNAIVFKGLWEKTFKDEDTQAMPFRVTEQESKPVQMMYQIGLFRVASMASEKMKILELP
FASGTMSMLVLLPDEVSGLEQLESIINFEKLTEWTSSNVMEERKIKVYLPRMKMEEKYNLTSVLMAMGITDVFSSSANLSGIS
SAESLKISQAVHAAHAEINEAGREVVGSAEAGVDAASVSEEFRADHPFLFCIKHIATNAVLFFGRCVSPGGGGSEQKLISEED
LGGGGSnalcgvafglgvlgtimgivfflcsqrpcsgd

>H2-Mb-OVA short

maalwllllvlsldcmgaGGGGGGSADQARELINSWVESQTNGIIRNVLQPSSVDSQTAMVLVNAIVFKGLWEKTFKDEDTQA
MPFRVTEQESKPVQMMYQIGLFRVASMASEKMKILELPFASGTMSMLVLLPDEVSGLEQLESIINFEKLTEWTSSNVMEERKI
KVYLPRMKMEEKYNLTSVLMAMGITDVFSSSANLSGISSAESLKISQAVHAAHAEINEAGREVVGSAEAGVDAASVSEEFRAD
HPFLFCIKHIATNAVLFFGRCVSPGGGGSEQKLISEEDLGGGGSvkvsvsaatlglgfiifcvgffrwrkshsssytplsgst
ypegqh

>H2-Mb-OVA long

maalwllllvlsldcmgaggfvahvestcvlddagtpqdftycvsfnkdllacwdpdvgkivpcefgvlypwaenfsrilnke
esllqrlqnglldcashtqpfwnalthrtrapsvrvaqttpfntrepvmlacyvwgfypadvtitwmkngqlvpshsnkekta
qpngdwtyqtvsylaltpsygdvytcvvqhsgtsepirgdwtpglspiqtvkGGGGSADQARELINSWVESQTNGIIRNVLQP
SSVDSQTAMVLVNAIVFKGLWEKTFKDEDTQAMPFRVTEQESKPVQMMYQIGLFRVASMASEKMKILELPFASGTMSMLVLLP
DEVSGLEQLESIINFEKLTEWTSSNVMEERKIKVYLPRMKMEEKYNLTSVLMAMGITDVFSSSANLSGISSAESLKISQAVHA
AHAEINEAGREVVGSAEAGVDAASVSEEFRADHPFLFCIKHIATNAVLFFGRCVSPGGGGSEQKLISEEDLGGGGSvkvsvsa
atlglgfiifcvgffrwrkshsssytplsgstypegqh

>H2-Oa-OVA short

mvlfvelvpvlltamsflsprgvraikGGGGSADQARELINSWVESQTNGIIRNVLQPSSVDSQTAMVLVNAIVFKGLWEKTF
KDEDTQAMPFRVTEQESKPVQMMYQIGLFRVASMASEKMKILELPFASGTMSMLVLLPDEVSGLEQLESIINFEKLTEWTSSN
VMEERKIKVYLPRMKMEEKYNLTSVLMAMGITDVFSSSANLSGISSAESLKISQAVHAAHAEINEAGREVVGSAEAGVDAASV
SEEFRADHPFLFCIKHIATNAVLFFGRCVSPGGGGSEQKLISEEDLGGGGSietlicglglvlglmgcllgtvlmitgtrrps
irr

>H2-Oa-OVA long

mvlfvelvpvlltamsflsprgvraikadhmgsygpafyqsydasgqfthefdgeqifsvdlkneevvwrlpefgdfahsdfq
sglmsismikahldilversnrtravsvpprvtvlpktrvelgkpnvlicivddifppvinvtwlrnsqpitkgvaqtsfysq
pnhrfrkfhyltfvpsaedvydckvehwgldtpllqhwepqvltpppdtieGGGGSADQARELINSWVESQTNGIIRNVLQPS
SVDSQTAMVLVNAIVFKGLWEKTFKDEDTQAMPFRVTEQESKPVQMMYQIGLFRVASMASEKMKILELPFASGTMSMLVLLPD
EVSGLEQLESIINFEKLTEWTSSNVMEERKIKVYLPRMKMEEKYNLTSVLMAMGITDVFSSSANLSGISSAESLKISQAVHAA
HAEINEAGREVVGSAEAGVDAASVSEEFRADHPFLFCIKHIATNAVLFFGRCVSPGGGGSEQKLISEEDLGGGGSietlicgl
glvlglmgcllgtvlmitgtrrpsirr

>H2-Ob-OVA short

mgagrapwvvallvnlmrldsfmiegrdGGGGSADQARELINSWVESQTNGIIRNVLQPSSVDSQTAMVLVNAIVFKGLWEKT
FKDEDTQAMPFRVTEQESKPVQMMYQIGLFRVASMASEKMKILELPFASGTMSMLVLLPDEVSGLEQLESIINFEKLTEWTSS
NVMEERKIKVYLPRMKMEEKYNLTSVLMAMGITDVFSSSANLSGISSAESLKISQAVHAAHAEINEAGREVVGSAEAGVDAAS
VSEEFRADHPFLFCIKHIATNAVLFFGRCVSPGGGGSEQKLISEEDLGGGGSsyswkkilsgaavfllglivflvgvvihlka
qkasvetqpgneasreslhsqp

>H2-Ob-OVA long

mgagrapwvvallvnlmrldsfmiegrdspenfviqakadcyftngtekvhllvrfifnleeylhfdsdlgmfvaltelgepd
adqwnkrldlletsraavnmvcrqkyklgapftvernvppevtvypertpllqqhnlllcsvtgfypgdisvkwfrngqeers
gvmstglvrngdwtfqttvmlemipelgdiysclvehpgllrpvsvawmaqseysGGGGSADQARELINSWVESQTNGIIRNV
LQPSSVDSQTAMVLVNAIVFKGLWEKTFKDEDTQAMPFRVTEQESKPVQMMYQIGLFRVASMASEKMKILELPFASGTMSMLV
LLPDEVSGLEQLESIINFEKLTEWTSSNVMEERKIKVYLPRMKMEEKYNLTSVLMAMGITDVFSSSANLSGISSAESLKISQA
VHAAHAEINEAGREVVGSAEAGVDAASVSEEFRADHPFLFCIKHIATNAVLFFGRCVSPGGGGSEQKLISEEDLGGGGSsysw
kkilsgaavfllglivflvgvvihlkaqkasvetqpgneasreslhsqp
